# Repeatability, reproducibility and consistency of horse shape data and its association with linearly described conformation traits in Franches-Montagnes stallions

**DOI:** 10.1371/journal.pone.0202931

**Published:** 2018-08-27

**Authors:** Annik Imogen Gmel, Thomas Druml, Katrin Portele, Rudolf von Niederhäusern, Markus Neuditschko

**Affiliations:** 1 Agroscope–Swiss National Stud Farm, Avenches, Switzerland; 2 Institute of Genetics, Vetsuisse Faculty, University of Bern, Bern, Switzerland; 3 Institute of Animal Breeding and Genetics, Veterinary University Vienna, Vienna, Austria; 4 Equine Sciences Faculty, Veterinary University Vienna, Vienna, Austria; University of Illinois, UNITED STATES

## Abstract

Linear description (LD) of conformation traits was introduced in horse breeding to minimise subjectivity in scoring. However, recent studies have shown that LD traits show essentially the same problems as traditionally scored traits, such as data converging around the mean value with very small standard deviations. To improve the assessment of conformation traits of horses, we investigated the application of the recently described horse shape space model based upon 403 digitised photographs of 243 Franches-Montagnes (FM) stallions and extracted joint angles based on specific landmark triplets. Repeatability, reproducibility and consistency of the resulting shape data and joint angles were assessed with Procrustes ANOVA (Rep) and intra-class correlation coefficients (ICC). Furthermore, we developed a subjective score to classify the posture of the horses on each photograph. We derived relative warp scores (PCs) based upon the digitised photos conducting a principal component analysis (PCA). The PCs of the shapes and joint angles were compared to the posture scores and to the linear description data using linear mixed effect models including significant posture scores as random factors. The digitisation process was highly repeatable and reproducible for the shape (Rep = 0.72–0.99, ICC = 0.99). The consistency of the shape was limited by the age and posture (p < 0.05). The angle measurements were highly repeatable within one digitiser. Between digitisers, we found a higher variability of ICC values (ICC = 0.054–0.92), indicating digitising error in specific landmarks (e.g. shoulder point). The posture scores were highly repeatable (Fleiss’ kappa = 0.713–0.857). We identified significant associations (p_(X_^2^_)_ < 0.05) with traits describing the withers height, shoulder length and incline, overall leg conformation, walk and trot step length. The horse shape data and angles provide additional information to explore the morphology of horses and therefore can be applied to improve the knowledge of the genetic architecture of LD traits.

## Introduction

Since the early stages of their domestication, horses have been selectively bred to produce offspring with adequate behavioural docility, overall health and a conformation adapted to their intended use (e.g. agriculture, transport, sports, leisure) [1]. Nowadays most breeding associations assess conformation traits by visual inspection and measure morphological traits, which are assumed to be associated with breed type, overall health, aesthetics or performance ability. Selection decisions are commonly based on such preliminary traits assessed before the animals start being used competitively. This way, selection intensity can be increased and the generation interval shortened. Initially, the evaluation of the horses’ conformation was based on the judgement of experts whether an animal corresponded to certain breeding objectives [2]. Subjective scoring protocols tend to include a combination of traits to provide an overall score. The scoring scale, which represents a weighting scale from good to bad, is often not used efficiently (lower end not used at all) with bias towards the optimum. Such protocols are useful in a breeding competition because they easily allow for a final ranking. However, the results presently do not serve only for the final ranking of a competition; they are also used in more complex animal models to identify traits which can be successfully improved by selection (e.g. [3,4,5]). Compared to measurable traits (e.g. height at withers), judged conformation traits have shown lower heritabilities, which has often been attributed to the broad trait definition and the subjectivity of judging [5].

Many breeding associations have opted to replace judging by linear description (LD), a method that has been used in cattle breeding since the 1980s and that describes traits according to the biological scale with the mean phenotype as a reference [2,6]. Assessors describe the animal on a biological scale by comparing it to the population mean and not in relation to a breeding goal (“good” or “bad”) [2]. In the Franches-Montagnes horse (FM), the last native horse breed of Switzerland, 19 conformation and 5 locomotion traits of three year old horses presented in hand are linearly assessed by a group of judges during a one day field test. Stallions additionally have to pass a 40-day stationary test to be approved for the breeding program. Recent publications report an insufficient use of the LD scale within the FM breeding program, with LD scores still converging around the mean and having small standard deviations [7,8]. This problem is not unique to the FM breed. International publications about other European breeds (e.g. Dutch [9] and Swedish [10] Warmblood, Finnhorse and Standardbred [5] and Haflinger [11]) observed similar problems with LD data, which is also a possible reason that heritabilities remained essentially in the same range (h^2^ = 0.10–0.50) as with traditional scoring [2].

To improve the assessment of conformation traits, a new methodology was proposed by Druml et al. [12] and applied in studies comparing subjective classifier rankings to objective measurement of horses using geometric morphometrics (GM). This method traces the shape of a horse from a photograph using landmarks and semi-landmarks, which can be statistically compared to data pertaining to judgements for example. The main feature of GM is that it standardises all shapes by translating, rotating and superimposing them according to their mean configuration. This process creates new coordinates that can be used for further analyses and are independent of actual size, and simultaneously describe shape variation [13]. Geometric morphometrics are widely used in biological research questions, and often applied on images of static objects such as bones [14,15]. To date, GM was not applied in a breeding context of horses. The horse shape space has been successfully applied to analyse differences in shape data of Lipizzan horses and ratings from different judges could be visualised on modified photographs [16]. One of the difficulties in the studies using the horse shape space seemed to be the body posture of the horses in the photographs, especially the head and neck, which accounted for more than 40% of total shape data variation [12]. The stance variation problem has also previously been reported in other equine conformation studies, and may even explain the left/right asymmetries measured in horses [17,18]. Furthermore, there is no information on whether there are asymmetries between shapes taken from the left or right hand side or whether age has an effect on shape, as could be expected from other conformation studies [19]. Nevertheless, the influence of such effects on the digitisation process have not been evaluated in detail.

Photographs are reasonably easy to take and often publicly available. Combined with GM, digital images could allow for measurements that are more precise without overly disrupting the schedule of the breeding presentations. However, before proposing new measuring technologies, they need to be evaluated for repeatability, reproducibility, consistency and compared to the data that is currently available (e.g. LD). The aim of the study was to evaluate the image based horse shape data in terms of repeatability, reproducibility and consistency and to test the validity of this method to assess conformation of horses. Specifically, we compared the shapes based on the same photograph within and between persons applying the horse shape space model (repeatability and reproducibility) and simultaneously examined different photographs of the same horses to test for left/right asymmetries and differences due to age (consistency). Using a set of FM stallion photographs, we extended the current GM application of the model by additionally calculating angular measurements for available joints, and developed statistical procedures to minimize the effects of posture on existing photographs. To assess whether the new method provides an additional merit compared to the current applied LD of FM horses, we finally compared the image based shape data and joint angles against the available LD data.

## Material and methods

To investigate the application of the horse shape space model in the FM breed in comparison to currently used LD data, and to address specific questions such as repeatability and consistency of this method, we used a total of 403 photographs of 243 FM stallions born between 1964 and 2014 (median = 2004). The selected photographs corresponded to the open posture as described in Druml et al. [12]: the forelimb closest in the photograph standing vertically; the hoof of the other foreleg one to two hoof lengths behind the closer forelimb; cannon bone of the hind limb closest in the photograph nearly vertical, the opposing hind limb two or three hoof lengths in front of the closer hind limb, with the head and neck “presented in a natural way”. For the majority of the stallions, only one photograph was available in the archive of the Swiss National Stud Farm, which had been taken during the station test at three years of age for studbook documentation. Some older photographs could not be dated, and the horses were assumed to be older than 3 years.

To investigate the consistency of the shape over time (age) and situations (side and repeated photographs within the same year), 58 stallions owned by the Swiss National Stud Farm, aged between 3 and 24 years old, were specifically photographed for this study from the left and right hand side in 2016 under the permit VD 3096. A sample of 23 stallions (among the 58) from the Swiss National Stud Farm were photographed multiple times on each side, which resulted in a total of 83 side comparisons overall (n_shapes_ = 166). Due to the suitability of the archive photographs, only 42 horses had a complete dataset for age comparisons between the stallions at the station test (all three years of age) and older (in 2016, 3 to 22 years of age, mean 10.38 years).

### Digitisation

We used a shape model to extract the shape information from photographs, which is composed of the outline of the horse and 31 additional somatometric landmarks [12]. The semi-landmarks from the curves were placed at equal distances within each curve and transformed to landmarks using tpsDig2 [20] and tpsUtil [20]. The final horse model comprised 246 landmarks (31 somatometric and 215 semi-landmarks) [12]. All images were mirrored in the same direction prior to digitising to limit the effect of measurer biasness (i.e. all horses’ heads face to the left).

Each photograph was digitised thrice by one digitiser (primary digitiser) in a semi-randomised order (1,209 shapes in total) to level out digitising error and assess intra-digitiser repeatability. Digitisation involves placing landmarks and drawing outlines with the mouse cursor in a predefined order. To assess intra-repeatability of a less experienced digitiser and compare the reproducibility on the same photograph between different digitisers (inter-digitiser repeatability), 62 photographs were randomly selected and digitised thrice by a second person and amounted to 179 shapes for the second digitiser after removing shapes showing errors in the order of the landmark placements. In total, 1,388 shapes over two digitisers with 341,448 two-dimensional landmarks were extracted for further analysis through tpsDig2 [21] and tpsUtil [20].

**Fig 1:**
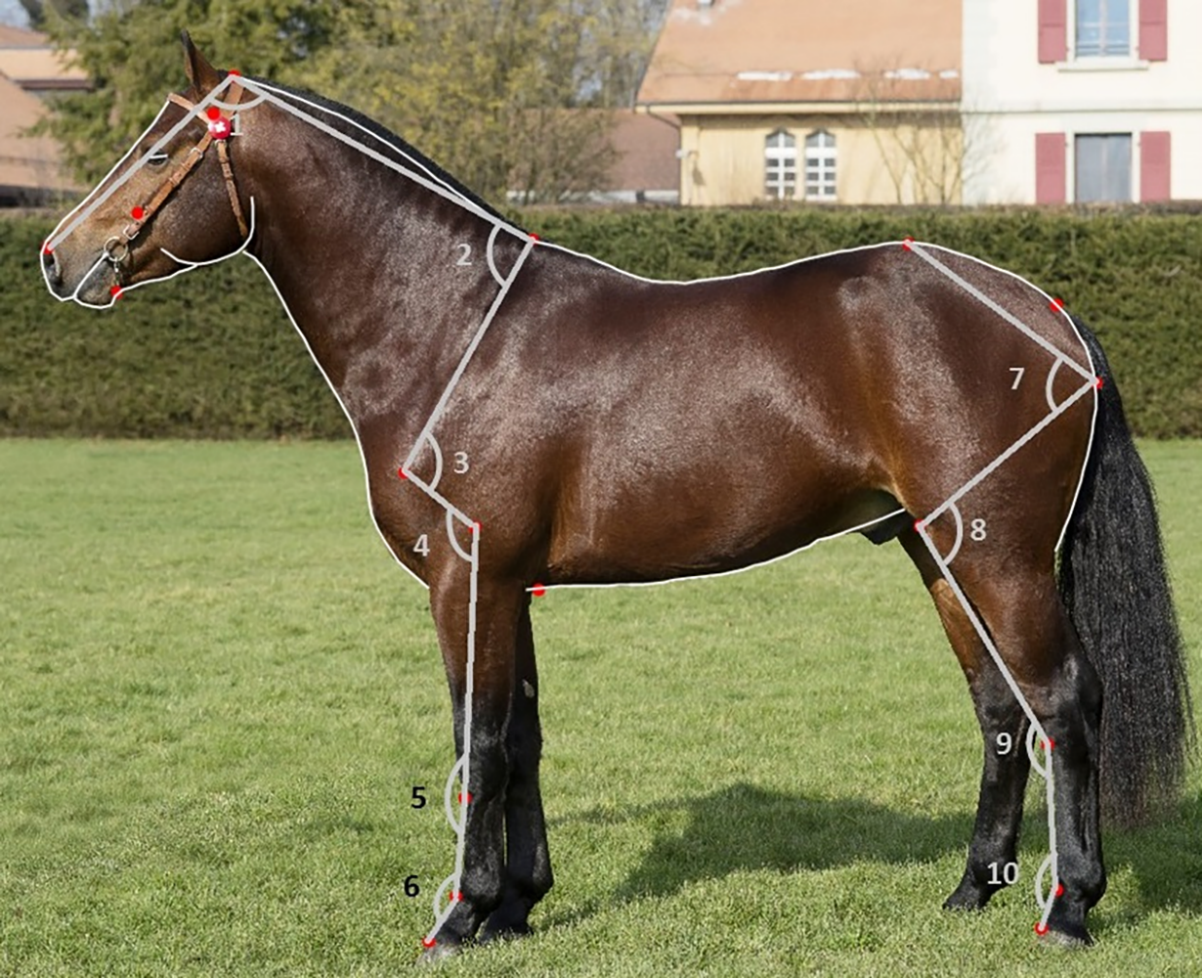
Example of the applied horse shape model with both curves and angles derived from landmarks.

In addition to the full shape, we calculated angles for available joints (Fig 1): the poll angle (1), neck-shoulder blade angle (2), shoulder joint angle (3), elbow joint angle (4), carpus angle (5), fore fetlock joint angle (6), hip joint angle (7), stifle joint angle (8), hock angle (9) and hind fetlock joint angle (10). Angles were extracted from the raw coordinates using basic trigonometry as implemented in statistical software R [22]. For each angle, the central landmark coordinates were set to 0, and the other two landmarks defining the angle were reconfigured by subtracting the central coordinates from them (x and y). We can then solve for the angle between the two new coordinates by using a trigonometric formula (see S1 Text). The specific landmarks defining each angle (see S1 Table) were selected from the original shape model [12].

### Processing of geometric morphometric data

The raw coordinates generated with the digitising tool tpsDig2 [21] depend on the distance of the horse from the camera and the pixel resolution of the digital image. In addition, the size of the animal itself may have an effect when comparing different horses. Thus, unlike for the angle measurements, the raw coordinates need to be normalised to be comparable across the whole dataset when comparing shapes. The most common approach is the Generalised Procrustes analysis (GPA), generating a new reference system, which scales, rotates and centres every shape according to the mean configuration of the sample [12], eliminating non-shape variation. The centroid of every shape (the mean of all x and y coordinate values) is translated on the origin, centring each shape. Size is normalised by dividing all x and y coordinates by the centroid size, which is the square root of the sum of all squared distances from each landmark to the centroid. The differences of object position on the images are normalised by minimising the squared differences between homologue landmarks (Procrustes distance). We extracted the Procrustes distance and the centroid size with the geomorph package as implemented in R [23] for repeatability, reproducibility and consistency tests.

### Repeatability of shape measurements

We evaluated the repeatability of the digitisation process (i.e. the measurement error) using two different approaches, by comparing centroid size and Procrustes distance. We analysed the repeatability of the centroid size of each normalised shape (the mean of all x and y coordinate values) using the intraclass correlation coefficient (ICC) with the ICC R package [24]. The interpretation of these values is based on Cicchetti [25]: an ICC under 0.40 is considered as poor, 0.41 to 0.59 as fair, 0.60 to 0.74 as good and 0.75 to 1.00 as excellent.

Secondly, we used the Procrustes distance as a measure to describe variance in the shapes. Procrustes ANOVA uses the Procrustes distance of the normalised coordinates as sums of squares and any independent variable as “treatment” effect in a traditional ANOVA sense. The relative amount of shape variation can thus be attributed to the independent variables, and significance is defined by the F-ratio and its statistic. To test the repeatability, we used the photograph as independent variable input; meaning all variation that is not explained by the differences between the photographs is due to digitising bias within each photograph (i.e. the digitising error). The repeatability based on the Procrustes distance (Rep) of the digitising process was calculated from the ANOVA results using a specific formula (see S2 Text) [26].

Furthermore, we assessed the intra-repeatability for the primary digitiser on all shapes (403 photographs x 3 repeats = 1,209 shapes) and for a second digitiser on the random subset (62 photographs x 3 repeats = 186 shapes). Given the seven excluded shapes among the triplets of repeats for the second digitiser, Rep was calculated by approximating n_repeats_ by the number of third repeats divided by the total number of photographs times three (n_repeat,approx_ = 2.66). We re-evaluated Rep of the primary digitiser on the same sample as the secondary digitiser to compare the ability to repeatedly digitise the same photograph accurately and assess potential sampling bias.

### Reproducibility of shape measurements

We calculated the mean x-y coordinates for each photograph out of the three repeats, reducing the number of shapes to one consensus shape per photograph and digitiser (403 from the first digitiser and 62 from the second digitiser, i.e. 465 shapes). Following a GPA of the consensus shapes, thereafter we evaluated the inter-repeatability between the two digitisers (reproducibility) by comparing the consensus shapes for the 62 randomly sampled horse digitised by both digitisers. The Procrustes ANOVA was calculated including the photograph as independent (treatment) variable; differences that are not due to the photograph itself are explained by differences in digitising. The ICC of the centroid size was calculated. The mean shape for the sample was plotted for each digitiser to identify discrepancies between the primary and secondary digitiser. For all subsequent analyses, the consensus shapes for each photograph digitised by the primary observer were used.

### Consistency of shape within an animal

We evaluated the consistency of the shape for different photographs of the same horse. Therefore, we compared the consensus shapes of 58 horses photographed on both sides (lateral view). We calculated the Procrustes ANOVA with the consensus shapes as the explanatory variable and the side and replicate (in case of multiple measures of the same horses) as independent variables. We also investigated the consistency of the horse shape over time, assessing whether age had an effect on shape by comparing the consensus shape of horses at three years old with the consensus shapes of the same horses taken in 2016 (42 horses, 3 to 22 years of age, mean 10.38 years, SD = 5.03, SE = 0.46, median age = 9). We computed a Procrustes ANOVA with the Procrustes distance as an explanatory, side and age as independent variables.

### Angles

For each angle, besides the descriptive statistics, we computed the ICC to estimate the digitising error. In order to test the consistency of each angle within the same horse (but different photographs) and to evaluate deviations between the consensus shapes of the two digitisers (inter-repeatability) we also used ICC.

### Effect of posture on the shape data and angle measurements

Given the previously reported influence of the horse’s posture on the retrieved shape variation [12], we aimed to describe the posture variation in our sample of photographs and to identify factors significantly influencing the shapes. We focused on previously described potential sources of variation, i.e. the height position of the head, neck posture and the position of the front and hind limb. In addition, we included three factors observed in our dataset in particular: whether the head was turned towards or away from the camera, whether the horse had its tail raised, and whether the horse was photographed in a straight way or if the front or hindquarters were closer to the camera (coplanarity). We further defined a subjective scoring system for each of these described factors based on the 403 photographs of our sample (see S3 Table) and tested the repeatability by comparing the classifications using an ICC and Fleiss’ kappa statistic (calculated with the irr R package [27]).

The 246 coordinates for each of the 403 consensus shapes (digitised by the primary investigator) were normalised using a GPA. We used a graphical method to exclude extreme outliers of digitised shapes falling over the upper quartile Procrustes distance from the mean shape of the sample (plotoutliers in the geomorph R package[23]). After exclusion of the outliers, the remaining consensus shapes were again normalised using GPA. We retained the relative warp scores (principal components (PCs) of the partial warp matrix) explaining the main variation in the data using a principal component analysis (PCA). The first five PCs were interpreted as meaningful shape data based on the inflection point on the scree plot displaying the percentage variance of each of the 492 components (246 x-y coordinates).

The effects of the posture factors on the shape data (PC1 to PC5 and each angle measurements) were assessed with a linear mixed effect model including the posture factors as fixed effects and the year of birth (YOB) and age category (3 year old vs older) as random effects. The significance of each shape data variable was evaluated in a multilevel approach comparing the full model versus the full model minus each tentatively significant fixed variable with a maximum likelihood function [28]. We also quantified the variation explained by the tested factors by calculating the marginal and conditional R^2^ using the R package piecewiseSEM [29]; the marginal R^2^ describes the proportion of variance solely explained by the fixed factors while the conditional R^2^ includes also the effects of the random factors [30]. In this study, we considered factors to have a *substantial* effect only when they are both statistically significant (p < 0.05) and with a conditional R^2^ > 0.3.

### Comparison of LD traits and novel derived shape variables

In order to compare the morphometric measurements to the currently applied LD method used to assess conformation in FM horses, we tested one consensus shape per horse against its LD data recorded at the age of three years during the stationary test. If more than one consensus shape was available for an individual, the one based on the photograph as a three-year old was preferentially used, as the photographs were taken approximately at the time the LD was performed. Stallions born before 1990 were excluded, as LD had not been introduced before that year.

We analysed the association of the shape data variables PC1 to PC5 and all angle measurements (poll, neck-shoulder blade, shoulder joint, elbow joint, carpus joint, fetlock joint in the forelimb, hip joint, stifle joint, hock, fetlock joint in the hind limb) with the LD data using linear mixed effect models. Each PC and angle variable was an explanatory variable in a separate model, and all 24 LD traits were fixed effects. The random effects were defined as follows: YOB and the age category (3 year old or older), and the corresponding previously defined posture factors with a substantial effect for each of the shape-derived variables. In the case of insufficient variation in the random structure, the irrelevant random factors were excluded to re-assess the model. The significance of each variable was evaluated in a multilevel approach following the same principle as describe above.

## Results

### Shape–repeatability and reproducibility

**Fig 2:**
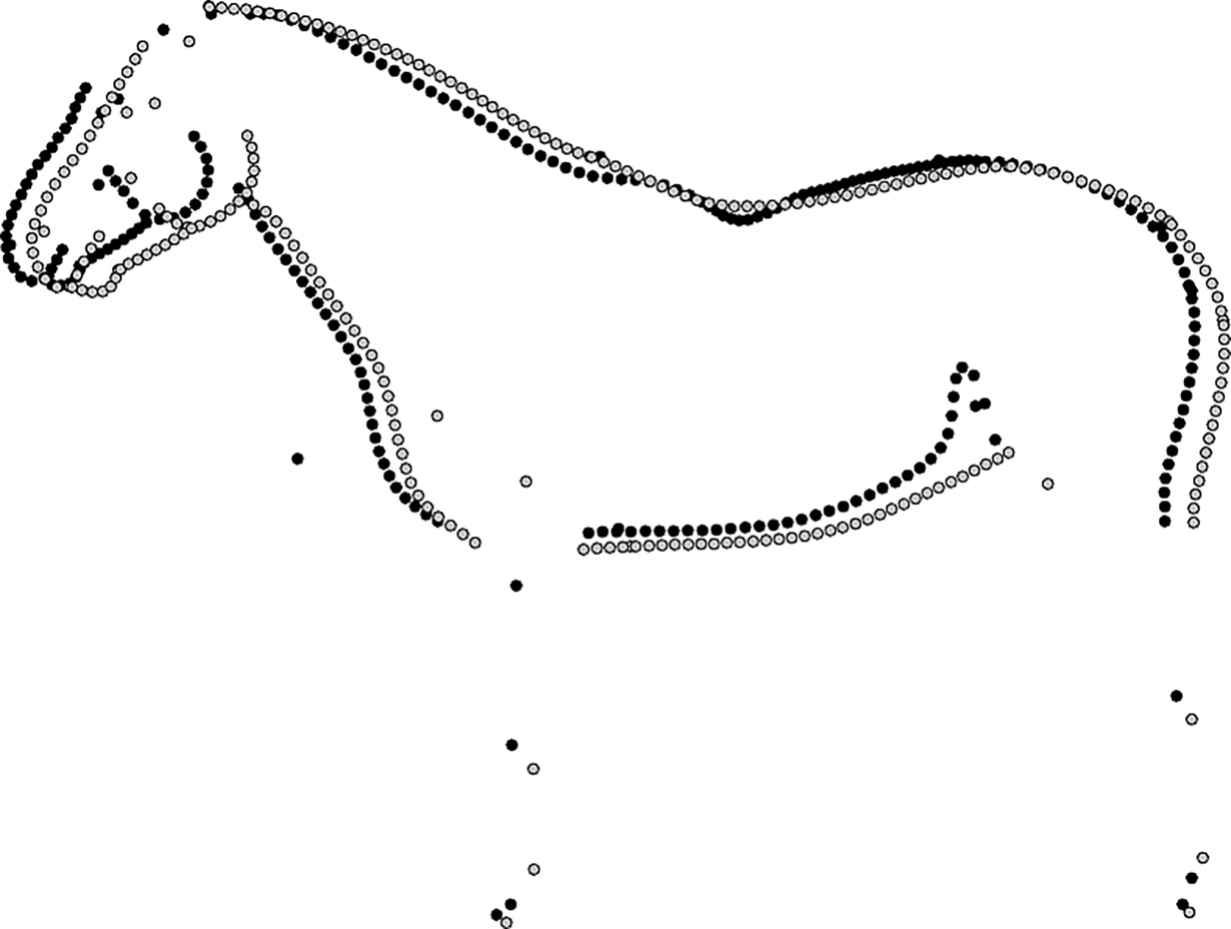
Comparison of the means from the common dataset between first digitiser (light grey) and second digitiser (black) using 62 shapes per digitiser.

For the main digitizer, the Rep (based on the Procrustes distance) and the ICC (based on the centroid size) were very high (0.99 for 1,209 shapes from 403 photographs). The repeatability of the secondary digitiser was high (Rep = 0.87), and the centroid size ICC was very high (ICC = 0.99) for 62 photographs. The repeatability for the same subset for the primary digitiser was Rep = 0.94 (ICC = 0.99). The reproducibility between the two digitisers on the small subset was Rep = 0.72 with an ICC = 0.99. Visually, the areas differing most are the lower side of the belly, the gaskin, the head and the landmarks describing the shoulder and limbs (Fig 2).

### Shape—Consistency

**Fig 3:**
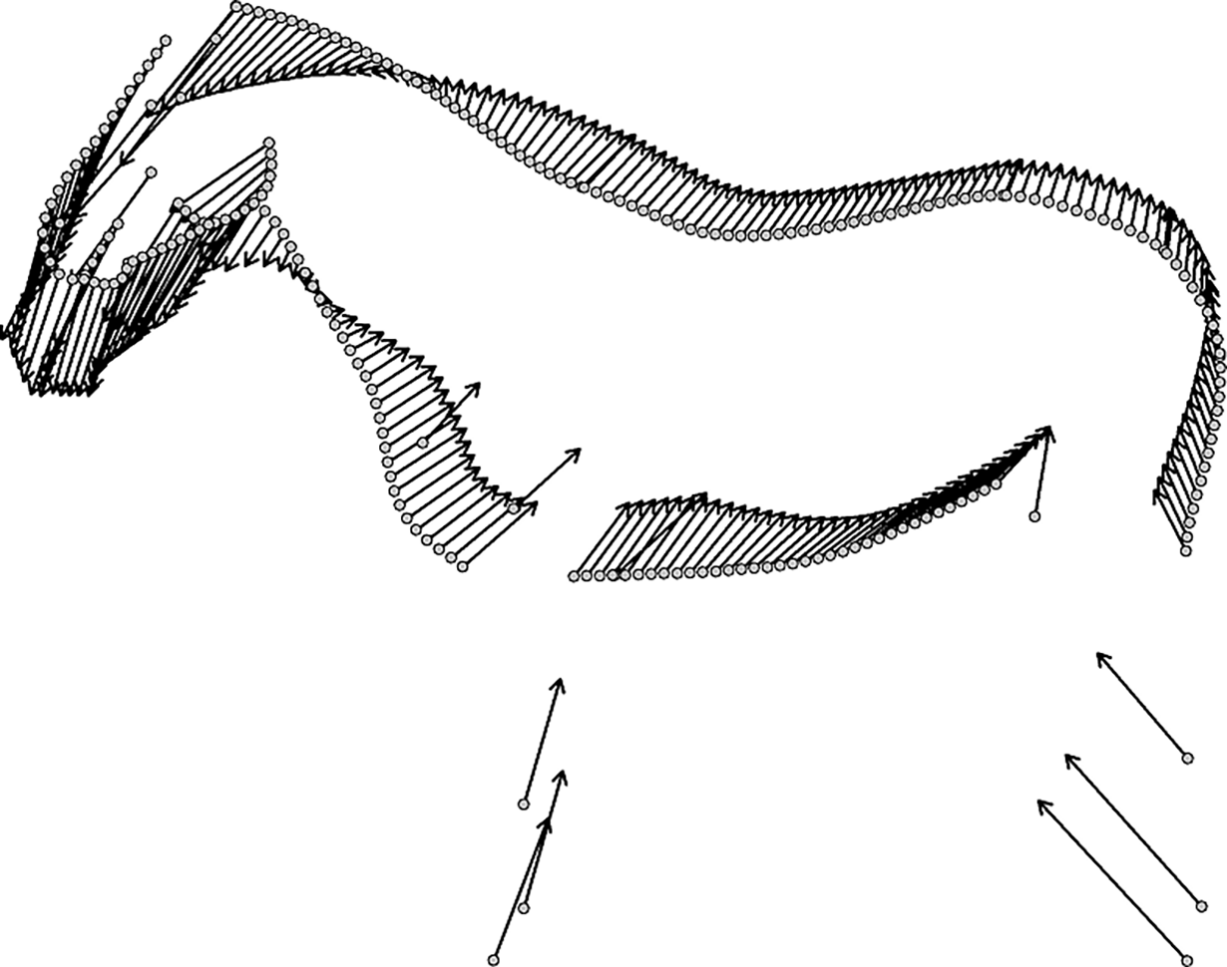
Comparison between the mean three-year old (n = 42) and mean older shape (n = 117). Arrows indicate differences from the young sample mean to the older sample mean.

Within the sample of 58 horses photographed from both sides, there was no significant difference between the left and right hand side based on the shape ANOVA (F_(1, 57)_ = 0.79, MSE = 0.00068, p = 0.49). There was a significant difference between the shapes of 42 horses at three years old and their shape later in life (F_(1, 158)_ = 35.62, MSE = 0.041, p = 0.001**). The side was not significant (F_(1,158)_ = 0.46, MSE = 0.00054, p = 0.37). Visually, strong age differences were observed in the shape of the neck, the gaskin, the underside of the belly, the breast and underside of the neck, the mandible, and the position of the limb landmarks (Fig 3).

### Angles

The ICC for angle measurements for the first digitiser ranged from poor to excellent (Table 1) and were higher within photographs (0.56 to 0.98) than between photographs of the same horse (0.071 to 0.82). The ICC between digitisers on the mean image ranged between 0.054 (shoulder joint angle) to 0.92 (poll angle). The angle with the highest range was also the one with the lowest ICC (shoulder). The descriptive results of the measured angles are summarised in S2 Table.

**Table 1:** ICC for repeatability (within the same photograph) and consistency (different photographs of the same horse) of the first digitiser, the second digitiser and the reproducibility between digitisers for the 10 measured angles.

	First digitiser (1209)		First digitiser (186)		Second digitiser (179)		Between digitisers (62)
	Repeatability	Consistency	Repeatability	Consistency	Repeatability	Consistency	Reproducibility mean photo
Poll (1)	0.98	0.56	0.99	0.84	0.98	0.82	0.92
Neck-shoulder blade (2)	0.90	0.50	0.95	0.63	0.91	0.56	0.71
Shoulder joint (3)	0.81	0.37	0.75	0.25	0.44	0.29	0.054
Elbow joint(4)	0.67	0.31	0.74	0.40	0.62	0.42	0.59
Carpus (5)	0.56	0.33	0.45	0.30	0.27	0.071	0.17
Fetlock joint forelimb (6)	0.76	0.42	0.76	0.46	0.59	0.50	0.57
Hip joint (7)	0.89	0.57	0.94	0.63	0.85	0.79	0.16
Stifle joint (8)	0.90	0.55	0.92	0.72	0.85	0.79	0.16
Hock (9)	0.76	0.42	0.90	0.75	0.67	0.46	0.75
Fetlock joint hind limb (10)	0.81	0.57	0.77	0.38	0.76	0.50	0.73

The number of photographs that were used in the comparisons are in parentheses.

### Postural effects

**Fig 4:**
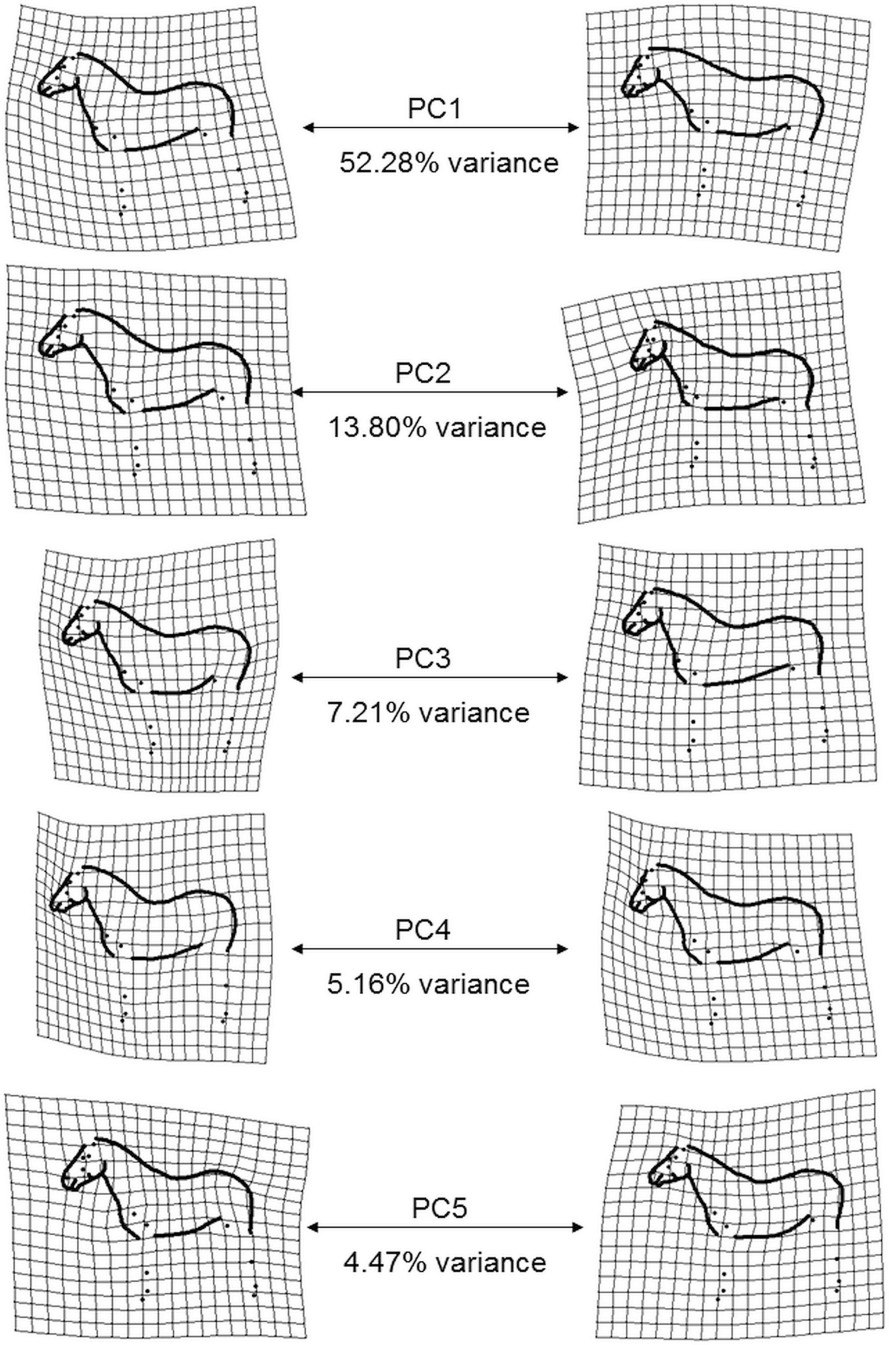
Representation of the extreme shapes describing the first five relative warp axes on warp grids.

From the visualisation plots of the first five PCs (displayed on their relative warp axes, Fig 4) we can observe relevant differences for the height position of the head (PC1), the poll angle (PC2), and the position of the front and hind limbs (PC3, PC4, PC5). The first five PCs account for 82.92% of total shape variation, with PC1 amounting to more than half of the total variation (52.28%). The scored postural variables showed good to excellent classifier repeatability (Fleiss’ к between 0.713 and 0.857, ICC between 0.768 and 0.923), with body position being the least repeatable (Table 2).

**Table 2:** Repeatability of all postural variables classified twice by the same rater, described by Fleiss’ kappa and the ICC.

Posture variable	Fleiss’ к	ICC
Head height	0.857	0.862
Head towards camera	0.765	0.852
Front limb	0.841	0.891
Hind limb	0.813	0.923
Body position	0.713	0.768
Tail	0.852	0.853

The main postural effects were defined as being statistically significant with a conditional R^2^ > 0.3. Only the effects that filled both conditions are reported in Table 3. The position of the front limb had no effect on any of the shape data. PC2 and PC5, as well as the shoulder joint, elbow joint, carpus, hock and both front and hind fetlock joint angles were not affected by any of the defined postural criteria. The conditional R^2^ was consistently and substantially higher than the marginal R^2^ for all models, indicating a better fit of the model when including the random structure.

**Table 3:** Effects of posture variables on shape data, described by marginal and conditional R2 and the Chi-squared significance, corrected for year of birth (YOB) and age category.

Shape-derived variable	Head height				Head camera				Hind limb				Body				Tail			
	Marg.R^2^ ^a^	Cond.R^2^ ^b^	Χ^2^	p	Marg.R^2^	Cond.R^2^	Χ^2^	p	Marg.R^2^	Cond.R^2^	Χ^2^	p	Marg.R^2^	Cond.R^2^	Χ^2^	p	Marg.R^2^	Cond.R^2^	Χ^2^	p
PC1	0.18	0.59	112.47	< 0.0001	0.015	0.63	11.10	0.0255	0.026	0.55	20.20	0.0004					0.026	0.59	10.73	0.001
PC3									0.24	0.47	148.81	< 0.0001								
PC4									0.25	0.42	116.81	< 0.0001								
Poll					0.052	0.39	32.17	< 0.0001									0.013	0.44	4.39	0.0362
Neck-shoulder blade	0.11	0.41	50.92	< 0.0001													0.038	0.38	11.34	0.0008
Hip joint^c^									0.18	0.37	96.52	< 0.0001								
Stifle joint									0.29	0.43	164.26	< 0.0001	0.089	0.37	59.06	< 0.0001				

^a^ marginal R^2^, describes the proportion of variance explained by the fixed factors

^b^ conditional R^2^, describes the proportion of variance explained by the fixed and the random factors

^c^ for the croup angle, the age category was excluded as a random factor

### Comparison of LD traits and shape data

Out of the 243 stallions with an available photograph, 191 were also linearly described once between 1990 and 2017 (see S4 Table for the definitions and descriptive statistics of the linear scores for the conformation and locomotion traits in the FM horse). Only five of the 24 LD traits (height of the withers, shoulder length, slope of the shoulder, overall quality of the legs, step length at walk and step length at trot) were significantly associated and meaningfully correlated with any of the shape data (Tables 4 and 5). These LD traits (except step length at walk and slope of the shoulder) were associated with more than one specific variable from the shape data. In more detail, stallions with a shape that had a very high neck position and front and hind limbs under the body were associated with less high withers (score 4 on LD 6). A smaller neck angle was also associated with less high withers (score 4 on LD 6). A larger croup angle was associated with a straighter shoulder slope (score 3 on LD 9) and cleaner, less puffy legs (overall quality of the legs, LD 18). PC2 and PC5, as well as the poll, shoulder joint, elbow joint, carpus, hock and both front and hind fetlock joint angles were not associated with any LD traits. The conditional R^2^ was consistently and substantially higher than the marginal R^2^ for all models, indicating a better fit of the model when including the random structure.

**Table 4:** Association between shape data with linearly described conformation variables described by marginal and conditional R^2^ and the Chi-squared significance.

Shape-derived variable	Height of the withers (LD 6)				Length of the shoulder (LD 8)				Slope of the shoulder (LD 9)				Overall quality of the leg (LD 18)				Random Structure
	Marg.R^2^ ^a^	Cond.R^2^ ^b^	Χ^2^	p	Marg.R^2^	Cond.R^2^	Χ^2^	p	Marg.R^2^	Cond.R^2^	Χ^2^	p	Marg.R^2^	Cond.R^2^	Χ^2^	p	
PC1	0.0018	0.75	4.18	0.041	0.041	0.75	4.21	0.040									Head height, head camera, hind limb, tail, age category, YOB
PC3	0.017	0.57	7.06	0.008	0.00070	0.44	8.18	0.004									Hind limb, age category, YOB
PC4													0.012	0.52	6.24	0.013	Hind limb, YOB
Neck-shoulder blade	0.0099	0.66	10.07	0.002	0.00045	0.65	8.73	0.003					0.013	0.65	8.24	0.004	Head height, tail, age category, YOB
Hip joint									0.010	0.53	7.07	0.008	0.012	0.54	5.43	0.020	Hind limb, YOB

^a^ marginal R^2^, describes the proportion of variance explained by the fixed factors

^b^ conditional R^2^, describes the proportion of variance explained by the fixed and the random factors

**Table 5:** Association between shape data and linearly described gait variables described by marginal and conditional R^2^ and the Chi-squared significance.

Shape-derived variable	Set length at walk (LD 19)				Step length at trot (LD 20)				Random structure
	Marg.R^2^ ^a^	Cond.R^2^ ^b^	Χ^2^	p	Marg.R^2^	Cond.R^2^	Χ^2^	p	
PC1	0.0053	0.75	5.12	0.024	0.0022	0.76	6.65	0.010	Head height, head camera, hind limb, tail, age category, YOB
PC4					0.0053	0.52	4.78	0.029	Hind limb, YOB

^a^ marginal R^2^, describes the proportion of variance explained by the fixed factors

^b^ conditional R^2^, describes the proportion of variance explained by the fixed and the random factors

## Discussion

### Shape–Repeatability and reproducibility

The repeatability for a given person digitising the same photograph was very high. The ICC based on the centroid size remained at 0.99 for all comparisons (intra-repeatability for each digitiser, reproducibility between the two digitisers) while Rep based on the Procrustes distance tended to be lower (0.72 to 0.99). The Procrustes ANOVA approach (Rep) has been reported to be more sensitive and thus more reliable than the centroid size approach, as the ICC based on the centroid size tends to be artificially high [31]. Based on the visualisation, a better definition of how to trace the abdomen and the placement of the landmarks for limbs and shoulder should increase the overall repeatability and reproducibility. On average, digitisation of one photograph takes between 10 and 15 minutes. The digitisation time decreases with training, but is hardly possible in less than 10 minutes per photograph. Considering the number of photographs, the manual digitisation process is time consuming and prone to errors. Therefore, the application of automation approaches through machine learning should be considered in future studies. Automation of landmark placement has been proposed for 3-dimensional data [32,33], and could thus potentially be implemented in the horse shape space model which is 2-dimensional and thus potentially simpler to compute. However, the number of landmarks in our model is larger compared to the aforementioned studies (246 vs 50 to 100), and the photographs are not fully standardised (different backgrounds, pixel resolution, posture of the horse) which may complicate the computation of the applied algorithms.

### Shape—Consistency

Contrary to other conformation studies [17,18], there was no significant asymmetry in the shape data between the left and right hand side of horses photographed at the same time, i.e. the side of the photograph was irrelevant and posture did not seem to have an effect on the shape. However, the mean shape of the three-year-old horses was significantly different from the mean shape of the same group of horses later in life. Differences can be noticed in the shape of the neck and the position of the limbs. This result is not unexpected, as the skeleton and the muscles are not fully developed in three-year-old horses, and stallions in particular often show increased neck circumference after several breeding seasons (due to age, genetic or hormonal factors) [34,35]. The observed differences may also be due to divergent postures between the photographs. We cannot entirely remove the postural variation when taking photographs of living horses, nor can we fully control the environment in which the horses develop. However, given the substantially higher conditional R^2^ compared to the marginal R^2^, we can mitigate these effects by accounting for postural differences by accurately classifying the photographs using the proposed classification method and include the age and postural effects in the statistical models.

### Angles

Repeatability of angle measurements based on three landmarks showed good to excellent repeatability within a triplet of photographs, but lower consistency when comparing all measurements within each animal, ranging from poor to good. The poll, neck-shoulder blade, stifle joint and hind fetlock joint angles in particular would be adequate traits for further genetic studies, as the repeatability (on the same image), the consistency (for a given animal on different images) and the reproducibility (between digitisers) was fair to high (good stability of the phenotype). However, especially poll and neck-shoulder blade angles also depend on the posture of the horse, which should be considered when using these traits in more complex models. The derived shoulder joint angle has the lowest repeatability of all computed angles. Furthermore, this angle showed no associations with either posture or linear traits. The centre point of this angle, the “point of the shoulder” is difficult to see in certain horses and should be marked after palpation if possible [19]. This may also explain the low validity of this particular angle and the fact that no relevant associations were found.

### Shape–Posture effects

Similarly to the other studies using the horse shape space model, the position of the neck, the head and the limbs were the largest source of variation in the shape data [12,16]. The height of the head had a highly significant effect on PC1, which was confirmed by the visual evaluation of the extreme deformation grids as well as from the conditional R^2^ of 0.59, one of the highest values in the study. It also had a highly significant effect on the neck-shoulder blade angle, which was expected as the neck-shoulder blade angle is defined by the position of the landmark on the crista nuchae on top of the head. PC1 was also associated with the position of the head towards the camera, the position of the tail and the position of the hind limb. Interestingly, posture effects concerning the head (head height and head towards camera) were associated with shape data variables together with the tail position (for PC1, the poll angle and the neck-shoulder blade angle). This could reflect a specific posture of a stallion in an “excited” state, i.e. with head held high and tail raised, sometimes with a very small poll angle.

The hind limb placement was most often associated with shape-derived variables (PC1, PC3, PC4, hip joint and stifle joint angles). The position of the hind limb may change the shape of the hind quarters, thus influencing overall shape. The angle of the hip joint may also be affected as the point of the buttocks and the stifle angle are displaced and digitisation of the landmarks is less precise. The association with the hock angle was also expected, as a hyperextension of the hind limb for example would increase the hock angle.

### Shape-derived variables and linear traits of FM stallions

The associations of several shape-derived variables (PC1, PC3, neck-shoulder blade angle) with height of the withers (and shoulder length) can be interpreted as a transition in breeding goals from a heavier draught horse to a riding horse, with the withers becoming more salient and less muscular to be more adapted to a saddle than a collar [35]. PC4 was also associated with LD18 (overall quality of the legs) and LD20, step length of the trot, which can be interpreted as a lighter type horse having clear legs and longer step length. Thus, PCs extracted from shape data are helpful to describe general conformation tendencies in an evolving breed. However, PCs could not be discriminately associated with a unique linear trait. This will make their interpretation in genetic studies contentious. Furthermore, the PCs will change depending on the sample, which makes them inconsistent as phenotypes. Another method to test if the LD data associate with shape variation is shape regression, as local deformations of the shapes (principal warps) are evaluated that are not dependent of the total sample. However, this is beyond the scope of the initial investigation. Measured angles such as shoulder joint, hip joint, hock and fetlock joints, which are size and time independent, were expected to associate with their corresponding linearly described traits slope of the shoulder, croup angle (for the hip joint), hock angle and fetlock angle as potential objective replacements of the subjective traits. Yet, they did not significantly associate, even with adequate statistical correction for postural effects. While in the case of the measured shoulder angle, this could be due to imprecisions in the shape data, for the other joint angles, which have shown both adequate repeatability and consistency, this discrepancy causes us to question the usability of LD data. The LD data of our study showed some traits with very narrow use of the scale (e.g. the fetlock angle, the hind limb muscling and the forelimb conformation), while some, such as the expression of the head, the correctness of gaits or the overall quality of the legs showed a broader range with a more adequate use of the scale. For the fetlock angle in particular, the LD data showed almost no variation (range of 3, SE = 0.025) while the joint angles showed a range of over 30° front and hind. In such cases, there can be no statistical associations between a measure and a description of seemingly the same trait, as while the measure shows adequate variation, the LD data shows almost none.

A smaller variation in a sample consisting only of stallions was expected due to the more severe selection criteria for approbation as breeding stallions. This was a constraint due to data availability, which focuses on stallions in the FM breed. However, adding mares and geldings in the shape sample may not change the overall results. The LD scores on the population also tend to converge around the mean or the extreme and show limited variation for the same LD traits [7], even if the variation in the measured sample would increase with the inclusion of mares and geldings with expected differences in morphology [34,35]. In previous studies in the Lipizzan horse breed [12,16], there was low reliability and concordance between raters on functional traits such as conformation of shoulder, withers, croup, legs as opposed to type-related traits, which may show the difficulty in the assessing process. In addition, in the FM breed, the record is based on one unique score only. It would be worth considering measuring these angles to improve the record of the variation of these functional traits in the population.

### Current limitations of shape data

One of the principal shortfalls of the shape space model is the dimensionality, i.e. that only one side of the horse is assessed, and that neither the front nor back view are included. This means that traits such as carpal varus/valgus, rotated cannon bone, the width of the chest and corresponding leg attachment (“base wide/narrow”) cannot be evaluated with the current application. Some three-dimensional systems are already available in the context of equine gait performance laboratories [17]. The setup, however, is cost-intensive and therefore limits the implementation in a routine breeding scheme. New camera systems are affordable from an infrastructure perspective, but require intense data processing after the measurement per se to extract the required information, and the initial difficulty of landmark placement is not fully addressed as some reference points are required to start the measuring process [36–38]. Overall, the results highlight a promising new measurement approach of conformation that is highly repeatable on a given photograph. The main difficulty that remains is the posture of the horse, which affects all aspects of the shape space and even the angle measurements. We have been able to mitigate the effects of the different postures by implementing a new scoring system for the photographs (S3 Table) that is highly repeatable.

### Future applications of the shape data in equine breeding

The new measures from the shape data are objective and depend mostly on the quality of the photograph. A photograph can be digitised directly after it has been shot, but we also have the opportunity to sample animals that may already be dead but of which photographs still exist in archives. In the future, automation of landmark placement would make the phenotyping less time intensive, more affordable, and more easily implementable in the field. Photographs can be taken during the field test and analysed throughout the day. Larger sample sizes would also allow us to be more stringent about selection of individual photographs and stabilise the variation in the shape and the PCs that adapt to the sample. Finally, we can use the shape space to visualise judging scores in a similar fashion to Druml and colleagues [12,16], to support training of judges of the breed, and to visually define the breeding objectives and the extremes of the scale needed for a more standardised LD model. Thus, the shape space model is a complementary tool to explore the morphology and genetic background of horses.

## Supporting information

S1 Text Trigonometric formula used for the extraction of angles. (DOCX)

S2 Text Formula used to evaluate the repeatability of the digitising process. (DOCX)

S1 Table Landmarks defining the different angle measurements used in the study. (DOCX)

S2 Table Descriptive statistics of the angle measurements. (DOCX)

S3 Table Classification of posture on photographs. (DOCX)

S4 Table Conformation and gait traits from the linear description of 191 FM stallions at three-years old. (DOCX)

S1 Fig Explanatory figure for TPS data of the horses. (TIF)

S1 Data TPS data of the horses. (TPS)
