# Supplementary material for: Repeatability, reproducibility and consistency of horse shape data and its association with linearly described conformation traits in Franches-Montagnes stallions
Source: PLoS One. 2018 Aug 27;13(8):e0202931. doi: 10.1371/journal.pone.0202931 (PMC6110498; doi:10.1371/journal.pone.0202931)
Supplement: S1 Text — (DOCX) [file pone.0202931.s001.docx]

S1 Text

Trigonometric formula used for the extraction of angles

$$\theta={cos}^{-1}\left( \frac{x\cdot y}{\left\| x \right\| \left\| y \right\|} \right)$$
