# Supplementary material for: Repeatability, reproducibility and consistency of horse shape data and its association with linearly described conformation traits in Franches-Montagnes stallions
Source: PLoS One. 2018 Aug 27;13(8):e0202931. doi: 10.1371/journal.pone.0202931 (PMC6110498; doi:10.1371/journal.pone.0202931)
Supplement: S2 Text — (DOCX) [file pone.0202931.s002.docx]

S2 Text

Formula used to evaluate the repeatability of the digitising process

$R=\frac{\frac{\left( {MS}_{among}-{MS}_{within} \right)}{n_{repeats}}}{{MS}_{within}+ \frac{\left( {MS}_{among}-{MS}_{within} \right)}{n_{repeats}}}$
