# Supplementary material for: Repeatability, reproducibility and consistency of horse shape data and its association with linearly described conformation traits in Franches-Montagnes stallions
Source: PLoS One. 2018 Aug 27;13(8):e0202931. doi: 10.1371/journal.pone.0202931 (PMC6110498; doi:10.1371/journal.pone.0202931)
Supplement: S1 Table — (DOCX) [file pone.0202931.s003.docx]

S1 Table

Landmarks defining the different angle measurements used in the study

| Angle name | x | origin | y |
| --- | --- | --- | --- |
| Poll (1) | Nostril (LM19) | Crista nuchae (LM2) | Highest point of the withers (LM3) |
| Neck-shoulder blade (2) | Crista nuchae (LM2) | Highest point of the withers (LM3) | Point of shoulder (LM12) |
| Shoulder joint (3) | Highest point of the withers (LM3) | Point of shoulder (LM12) | Approximate position of elbow joint (LM10) |
| Elbow joint (4) | Point of shoulder (LM12) | Approximate position of elbow joint (LM10) | Metacarpal tuberosity (LM11) |
| Carpus (5) | Approximate position of elbow joint (LM10) | Metacarpal tuberosity (LM11) | Metacarpophalangeal joint (LM15) |
| Fetlock joint of the forelimb (6) | Metacarpal tuberosity (LM11) | Metacarpophalangeal joint (LM15) | Coronet of the front hoof (LM16) |
| Hip joint (7) | Highest point of the croup (LM4) | Furthest point of the buttock (LM6) | Patella (LM8) |
| Stifle joint (8) | Furthest point of the buttock (LM6) | Patella (LM8) | Tarsometatarsal joint (LM7) |
| Hock (9) | Patella (LM8) | Tarsometatarsal joint (LM7) | Metatarsophalangeal joint (LM18) |
| Fetlock joint of the hind limb(10) | Tarsometatarsal joint (LM7) | Metatarsophalangeal joint (LM18) | Coronet of the hind hoof (LM17) |
