# Supplementary material for: Repeatability, reproducibility and consistency of horse shape data and its association with linearly described conformation traits in Franches-Montagnes stallions
Source: PLoS One. 2018 Aug 27;13(8):e0202931. doi: 10.1371/journal.pone.0202931 (PMC6110498; doi:10.1371/journal.pone.0202931)
Supplement: S2 Table — (DOCX) [file pone.0202931.s004.docx]

S2 Table

Descriptive statistics of the angle measurements

| Angle | Mean | SD | Median | Min | Max | Range |
| --- | --- | --- | --- | --- | --- | --- |
| Poll (1) | 104.72 | 4.52 | 105.08 | 92.36 | 117.84 | 25.48 |
| Neck-shoulder blade (2) | 82.32 | 6.21 | 82.16 | 64.11 | 99.47 | 35.36 |
| Shoulder joint (3) | 93.62 | 8.60 | 91.51 | 77.79 | 122.54 | 44.75 |
| Elbow joint (4) | 127.30 | 6.00 | 127.10 | 109.80 | 146.40 | 36.6 |
| Carpus (5) | 178.00 | 1.22 | 178.30 | 173.30 | 179.60 | 6.3 |
| Fetlock joint of the forelimb (6) | 151.40 | 3.91 | 151.20 | 138.20 | 172.10 | 33.9 |
| Hip joint (7) | 77.83 | 3.06 | 77.64 | 70.58 | 88.67 | 18.09 |
| Stifle joint (8) | 100.95 | 4.78 | 100.54 | 87.44 | 121.19 | 33.75 |
| Hock (9) | 153.30 | 2.21 | 153.10 | 147.00 | 159.80 | 12.8 |
| Fetlock joint of the hind limb (10) | 159.80 | 4.70 | 159.80 | 146.20 | 177.90 | 31.7 |
