# Supplementary material for: Repeatability, reproducibility and consistency of horse shape data and its association with linearly described conformation traits in Franches-Montagnes stallions
Source: PLoS One. 2018 Aug 27;13(8):e0202931. doi: 10.1371/journal.pone.0202931 (PMC6110498; doi:10.1371/journal.pone.0202931)
Supplement: S3 Table — (DOCX) [file pone.0202931.s005.docx]

S3 Tables

Classification of posture on photographs

Table S3.1

Subjective criteria for the classification of photographs

| **Criterion and scale** | **Code** |
| --- | --- |
| **Head height** | **1-3** |
| Tip of the nose above the lowest point of the back | 1 |
| Tip of the nose between the lowest point of the back and the attachment of the neck | 2 |
| Tip of the nose below the attachment of the neck | 3 |
| **Head position in relation to the camera** | **1-5** |
| Head turned away from the camera (lateral nostril wing almost indistinguishable, vascular groove of ventral mandibular border visible) | 5 |
| Head turned slightly away from the camera (zygomatic process of the frontal bone above the frontal bone) | 4 |
| Head facing straight ahead | 3 |
| Head turned slightly towards camera (opposite zygomatic process of the frontal bone visible) | 2 |
| Head turned towards camera (opposite medial nostril wing visible) | 1 |
| **Forelimb position** | **2-5** |
| In front of the body (front of the hoof in front of the breast) | 5 |
| Slightly in front of the body (front of the hoof in front of the attachment of the forelimb) | 4 |
| Straight (front of the hoof on the line with the attachment of the forelimb) | 3 |
| Front of the hoof in front of the withers but behind the attachment of the forelimb | 2 |
| **Hind limb position** | **1-5** |
| Hind limb under the body (front of the hoof in front of the stifle) | 5 |
| Hind limb slightly under the body | 4 |
| Cannon straight/perpendicular to ground | 3 |
| Slightly hyperextended hind limb | 2 |
| Hyperextended hind limb (front of the hoof behind the farthest point of the croup) | 1 |
| **Body position** | **1-5** |
| Hind quarters closer to the camera than forequarters (opposing point of buttock visible, breast visible through the front limbs) | 1 |
| Hind quarters closer to the camera than forequarters (breast visible through the front limbs) | 2 |
| Straight | 3 |
| Forequarters closer to the camera than hindquarters, (tail dock barely visible) | 4 |
| Forequarters closer to the camera than hindquarters, (tail dock barely visible, opposing forearm visible in front of the nearest forearm) | 5 |
| **Tail** | **1-2** |
| Relaxed tail | 1 |
| Fully raised tail (tail dock fully distinct from the croup) | 2 |

S3.2 Table

Head height

| 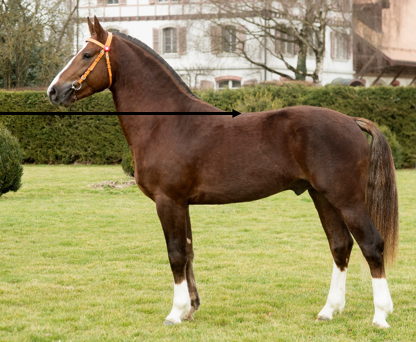 | 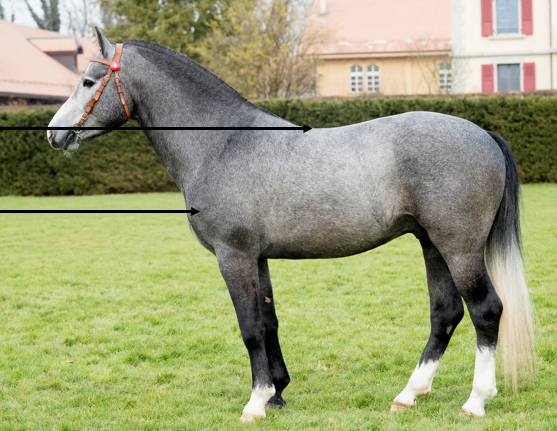 |
| --- | --- |
| Tip of the nose above the lowest point of the back (1) | Tip of the nose between the lowest point of the back and the attachment of the neck (2) |
| 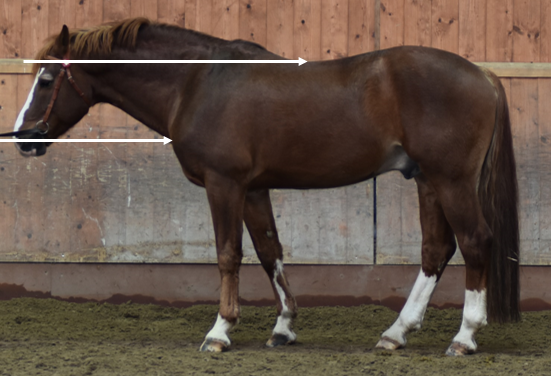 |  |
| Tip of the nose below the attachment of the neck (3) |  |

S3.3 Table

Head position in relation to the camera

| 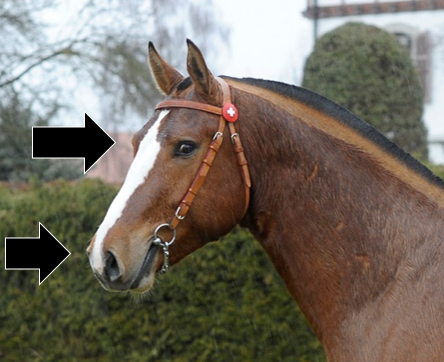 | 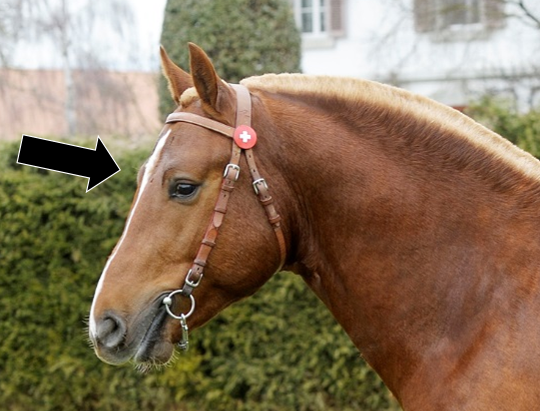 |
| --- | --- |
| Turned towards camera (1) | Turned slightly towards the camera (2) |
| 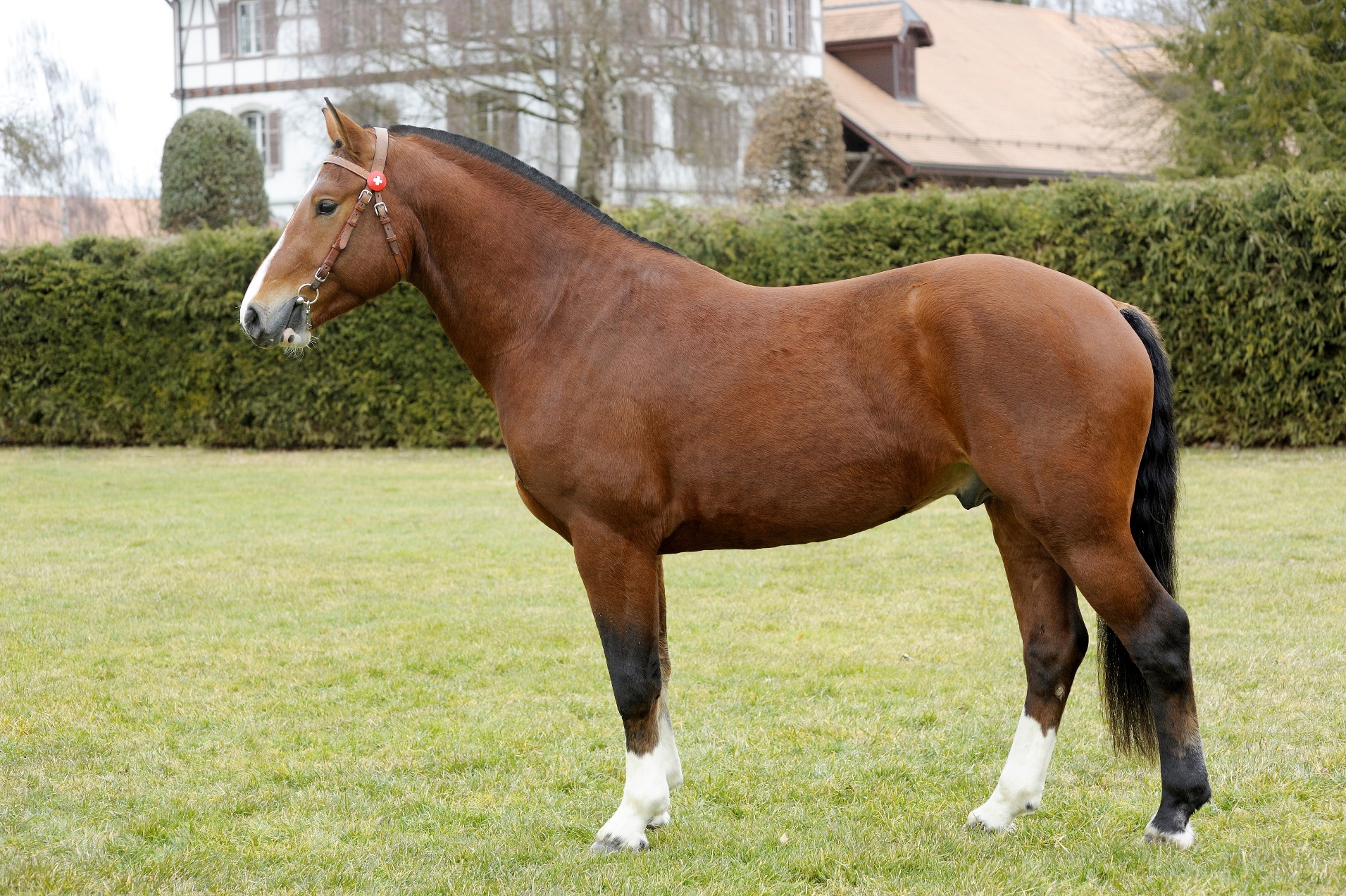 | 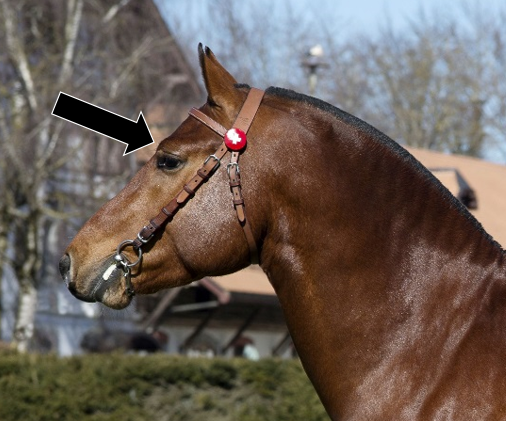 |
| Centered, facing straight ahead (3) | Turned slightly away from the camera (4) |
| 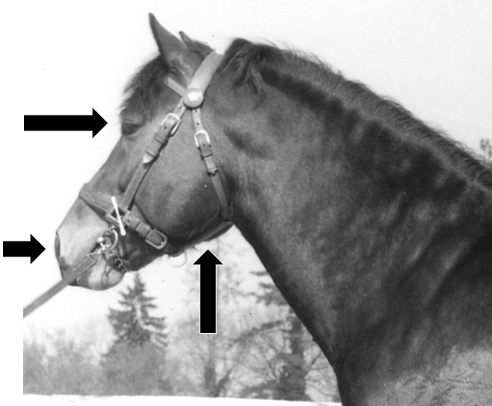 |  |
| Turned away from the camera (5) | |

S3.4 Table

Forelimb position

| 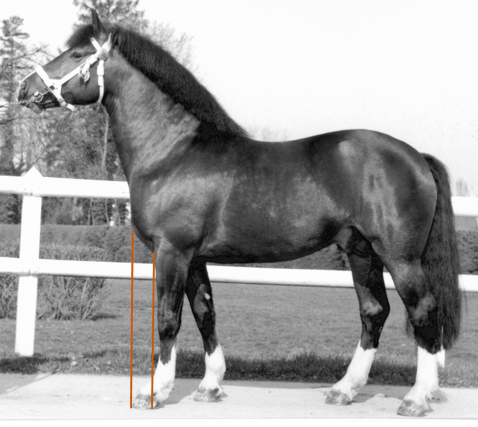 | 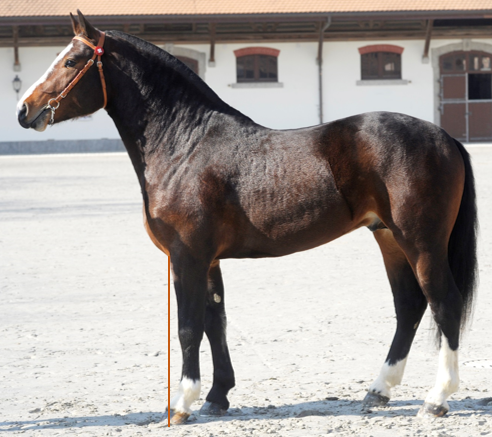 |
| --- | --- |
| In front of the body (front of the hoof in front of the breast) (5) | Slightly in front of the body (front of the hoof in front of the attachment of the forelimb) (4) |
| 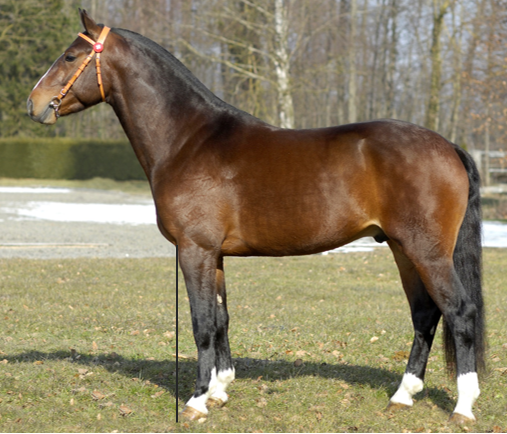 | 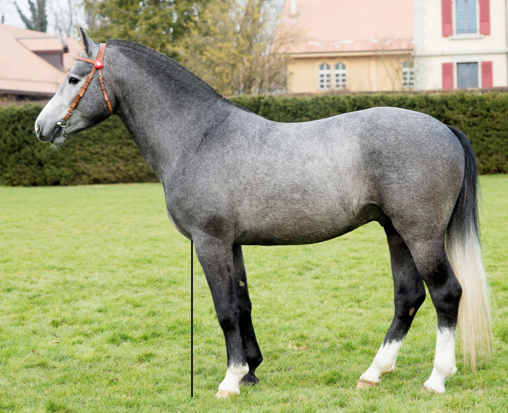 |
| Straight (front of the hoof on the line with the attachment of the forelimb) (3) | Front of the hoof behind the attachment of the forelimb (2) |

S3.5 Table

Hindlimb position

| 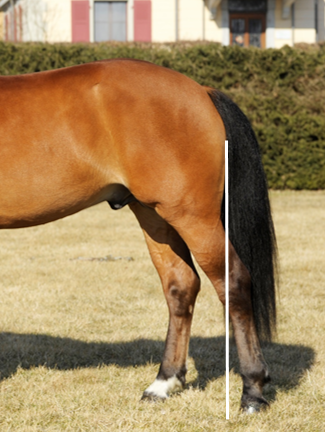 | 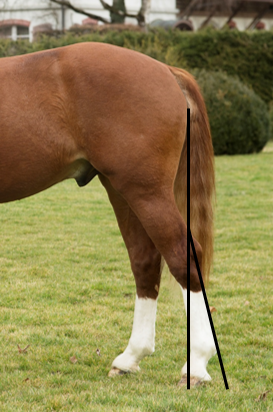 |
| --- | --- |
| Hyperextended hind limb (1) | Slightly hyperextended hind limb (2) |
| 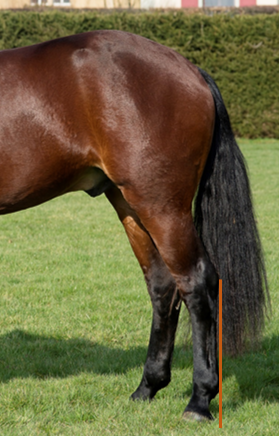 | 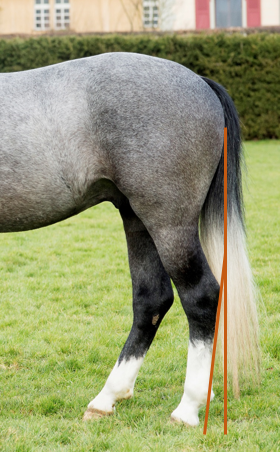 |
| Straight (3) | Slightly under the body (4) |
| 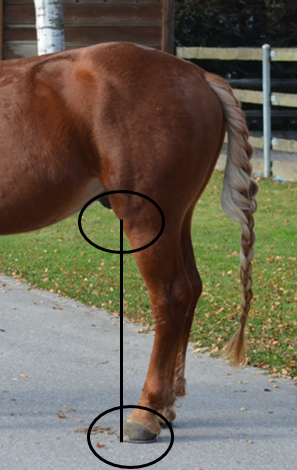 |  |
| Under the body (5) |  |

S3.6 Table

Body position

| 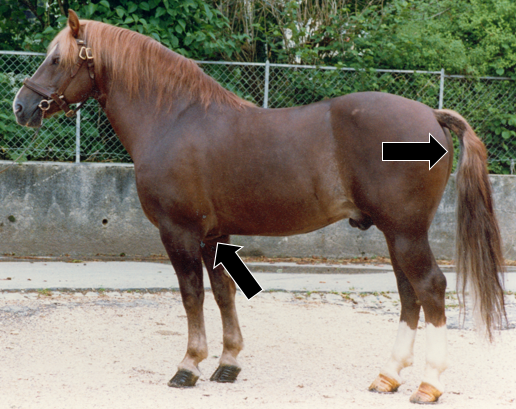 | 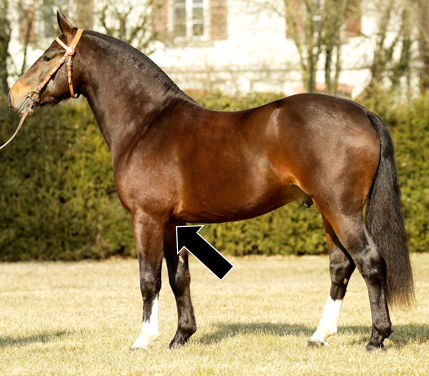 |
| --- | --- |
| Opposing point of buttock visible (1) | Hind quarters closer to the camera than forequarters (breast visible through the front limbs) (2) |
| 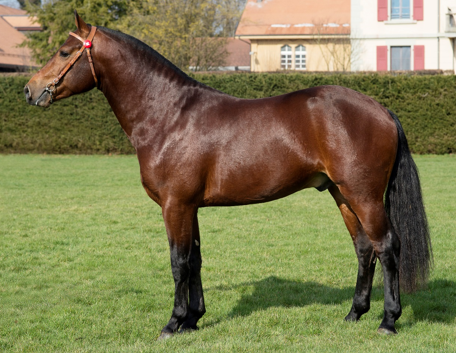 | 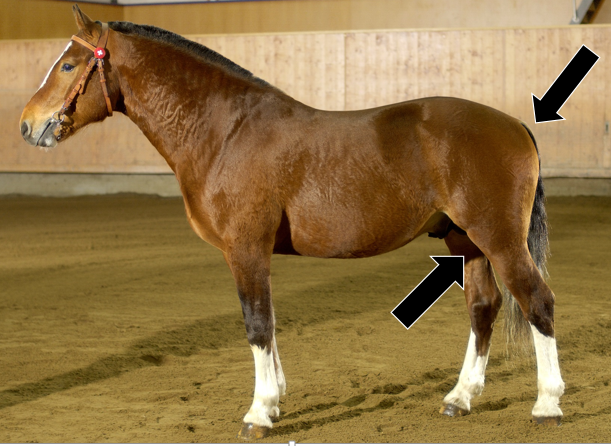 |
| Straight (3) | Forequarters closer to the camera than hindquarters*,* (tail dock barely visible) (4) |
| 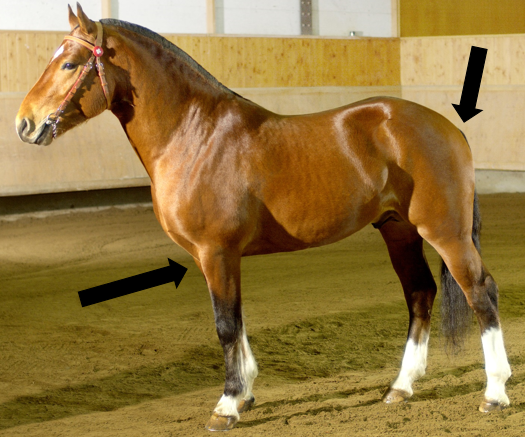 |  |
| Opposing forearm visible in front of the nearest forearm (5) |  |

S3.7 Table

Tail position

| 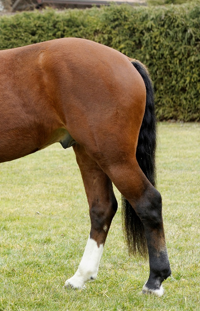 | 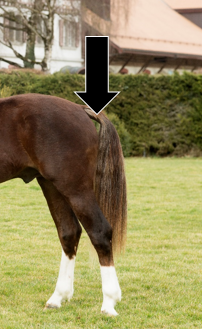 |
| --- | --- |
| Relaxed tail | Fully raised tail (tail dock fully distinct from the croup) |
