# Supplementary material for: Repeatability, reproducibility and consistency of horse shape data and its association with linearly described conformation traits in Franches-Montagnes stallions
Source: PLoS One. 2018 Aug 27;13(8):e0202931. doi: 10.1371/journal.pone.0202931 (PMC6110498; doi:10.1371/journal.pone.0202931)
Supplement: S4 Table — (DOCX) [file pone.0202931.s006.docx]

S4 Table

Conformation and gait traits from the linear description of 191 FM stallions at three-years old

| # | Trait | Extreme minimum | Extreme maximum | Mean | SD | SE | Median | Min | Max | Range |
| --- | --- | --- | --- | --- | --- | --- | --- | --- | --- | --- |
| 1 | Expression of the head | Expressionless | Expressive | 6.94 | 1.22 | 0.088 | 7 | 2 | 9 | 7 |
| 2 | Jaw-line | Heavy | Free | 5.96 | 1.14 | 0.083 | 6 | 3 | 9 | 6 |
| 3 | Length of the neck | Short | Long | 5.65 | 1.08 | 0.078 | 5 | 3 | 8 | 5 |
| 4 | Neck-chest junction | Low | High | 5.17 | 0.77 | 0.056 | 5 | 3 | 8 | 5 |
| 5 | Neck muscling | Lean | Massive | 5.36 | 0.73 | 0.053 | 5 | 4 | 8 | 4 |
| 6 | Height of the withers | Flat | Well-defined | 6.17 | 0.91 | 0.066 | 6 | 4 | 9 | 5 |
| 7 | Length of withers | Short | Long | 5.76 | 1.03 | 0.075 | 6 | 4 | 9 | 5 |
| 8 | Length of the shoulder | Short | Long | 6.00 | 0.85 | 0.061 | 6 | 4 | 8 | 4 |
| 9 | Slope of the shoulder | Upright | Sloping | 5.73 | 1.03 | 0.074 | 6 | 3 | 8 | 5 |
| 10 | Length of the back | Short | Long | 5.59 | 0.75 | 0.054 | 5 | 4 | 8 | 4 |
| 11 | Line of the back (top line) | Dipped | Roached | 4.63 | 0.67 | 0.048 | 5 | 3 | 7 | 4 |
| 12 | Length of the croup | Short | Long | 5.34 | 0.89 | 0.065 | 5 | 3 | 8 | 5 |
| 13 | Slope of the croup | Horizontal | Falling | 5.57 | 0.80 | 0.058 | 5 | 3 | 7 | 4 |
| 14 | Hind limb muscling | Weak | Strong | 5.16 | 0.50 | 0.036 | 5 | 4 | 7 | 3 |
| 15 | Forelimb conformation | Back at the knee | Over at the knee | 4.75 | 0.53 | 0.039 | 5 | 2 | 6 | 4 |
| 16 | Hock angle | Straight | Angled | 5.21 | 0.61 | 0.044 | 5 | 3 | 8 | 5 |
| 17 | Fetlock angle | Upright | Sloping | 5.06 | 0.34 | 0.025 | 5 | 4 | 7 | 3 |
| 18 | Overall quality of the legs | Puffy | Clean | 5.78 | 1.21 | 0.088 | 5 | 3 | 9 | 6 |
| 19 | Step length at walk | Short | Long | 6.85 | 0.93 | 0.067 | 7 | 4 | 9 | 5 |
| 20 | Step length at trot | Short | Long | 7.16 | 0.83 | 0.060 | 7 | 4 | 9 | 5 |
| 21 | Impulsion of the trot | Weak | Powerful | 7.03 | 0.89 | 0.064 | 7 | 5 | 9 | 4 |
| 22 | Suppleness of the trot | Stiff | Elastic | 6.96 | 0.94 | 0.068 | 7 | 4 | 9 | 5 |
| 23 | Correctness of gaits | Plaiting | Dishing | 4.92 | 0.75 | 0.054 | 5 | 2 | 9 | 7 |
| 24 | Type | Plain | True to type | 7.34 | 0.95 | 0.069 | 7 | 5 | 9 | 4 |
