# Supplementary figures and images for: Repeatability, reproducibility and consistency of horse shape data and its association with linearly described conformation traits in Franches-Montagnes stallions
Source: PLoS One. 2018 Aug 27;13(8):e0202931. doi: 10.1371/journal.pone.0202931 (PMC6110498; doi:10.1371/journal.pone.0202931)

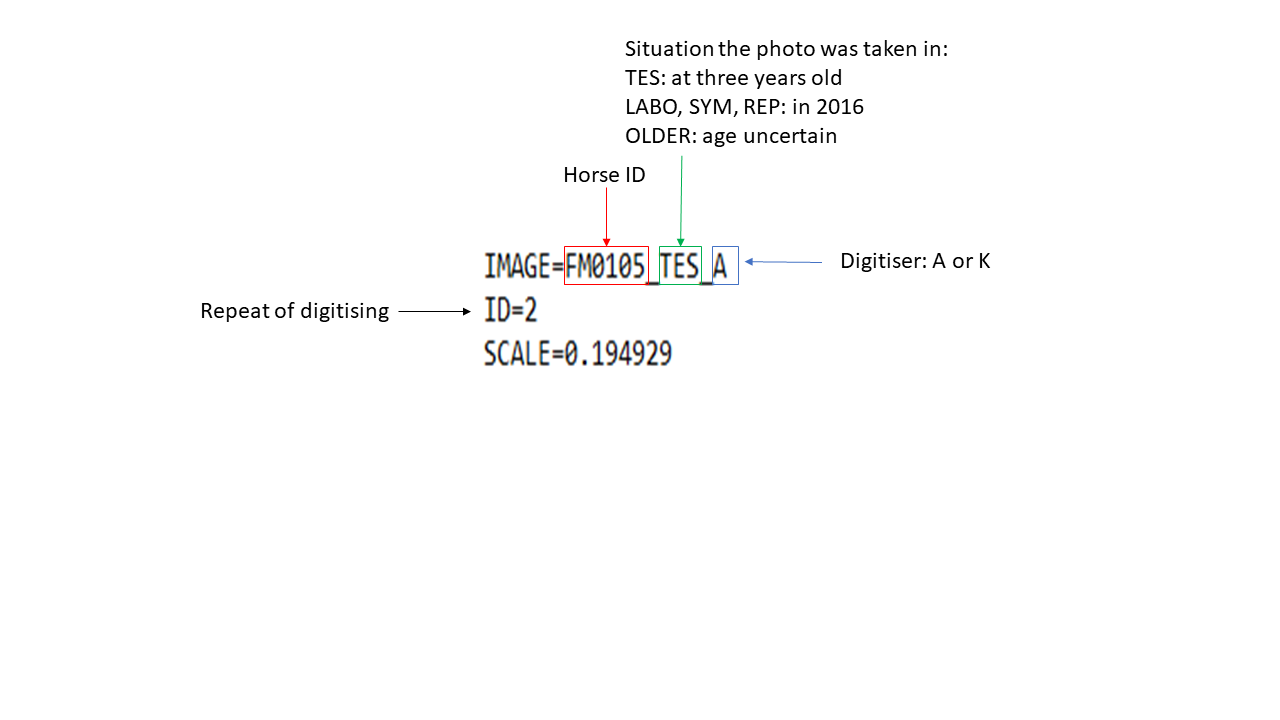

Supplement: S1 Fig — (TIF) [file pone.0202931.s007.tif]
